# Impaired Formation of Primary Cilia in Olfactory Neuronal Precursors Is Associated with Decreased Proliferation and Maturation in Individuals with Hyposmia

**DOI:** 10.3390/ijms26199435

**Published:** 2025-09-26

**Authors:** Salvador Alarcón-Elizalde, Alejandra Lora-Castellanos, Valeria Santillán-Morales, Miguel A. Reséndiz-Gachús, Rosa Estrada-Reyes, Julián Oikawa-Sala, Jesús Muñoz-Estrada, Lilian Mayagoitia-Novales, Luis A. Constantino-Jonapa, Cristina Martín-Higueras, Ángel Acebes, Gloria Benítez-King

**Affiliations:** 1 Laboratorio de Neurofarmacología, Instituto Nacional de Psiquiatría Ramón de la Fuente Muñiz, Mexico City 14370, Mexico; salalel@inpfr.gob.mx (S.A.-E.); ale.lora.orl@gmail.com (A.L.-C.); santllan.val.29@gmail.com (V.S.-M.); miguelgachus@gmail.com (M.A.R.-G.); oikawasala@inprf.gob.mx (J.O.-S.); bilogia0712@gmail.com (L.A.C.-J.); 2 Laboratorio de Fitofarmacología, Instituto Nacional de Psiquiatría Ramón de la Fuente Muñiz, Mexico City 14370, Mexico; restrada@inprf.gob.mx; 3 Department of Computational Biomedicine, Cedars Sinai Medical Center, Los Angeles, CA 90069, USA; josedejesus.munozestrada@cshs.org; 4 Departamento de Etología, Instituto Nacional de Psiquiatría Ramón de la Fuente Muñiz, Mexico City 14370, Mexico; mayagn@inprf.gob.mx; 5 Department of Basic Medical Sciences, Institute of Biomedical Technologies (ITB), University of La Laguna (ULL), 38200 Tenerife, Spain; cristinamh24@gmail.com

**Keywords:** hyposmia, olfactory sensory neurons, primary cilia, olfactory neuronal precursors

## Abstract

Smell dysfunction affects quality of life and is considered an early clinical sign of Alzheimer’s and Parkinson’s diseases. Olfactory loss increases with age and is associated with certain ciliopathies, a group of genetic disorders characterized by a wide spectrum of multisystemic disturbances. The dysfunction of mature olfactory sensory neurons (OSNs) in the olfactory neuronal pathway remains poorly understood. Previous evidence suggests that primary cilia proteins are involved in the maturation of olfactory sensory neurons (OSNs). In this study, we obtained olfactory neuronal precursors (ONPs) from the olfactory mucosa of young and older healthy volunteers who reported smell impairment (hyposmia) without neurological deficits or underlying airflow issues (conductive olfactory loss) and from normosmic individuals. In vitro analysis of ONPs showed that these cells can form primary cilia in normosmic individuals, while in hyposmic participants, there is a reduction in cilia frequency and a shorter length. In addition, ONPs from hyposmic individuals had a decrease in proliferation and cell differentiation. Our data indicate that alterations in molecular pathways related to primary cilia formation and the proliferation of ONPs lead to defects in neuronal maturation. These changes may hinder the differentiation of olfactory sensory neurons OSNs and contribute, at least in part, to olfactory loss.

## 1. Introduction

Olfaction is an ancient sense that connects organisms with their environment, allowing them to feed, mate, and survive by triggering innate responses, evolutionarily conserved as fight or flight, to face potentially dangerous situations [1].

Within the brain, odors trigger cognition, memories, and emotions. Therefore, olfactory dysfunction diminishes individual survival but also reduces their quality of life [2]. Olfaction loss is one early feature of neurodegenerative diseases such as Alzheimer’s (AD) and Parkinson’s (PD) [3]. Moreover, neuropsychiatric diseases such as schizophrenia, bipolar disorders, and major depression have also been associated with a decreased threshold of olfactory perception [4,5,6]. Importantly, olfactory impairments in psychiatric disorders are linked to a decrease in olfactory bulb volume and dysfunctional connectivity of brain regions related to odor processing [7]. One of the structures involved in this function is the entorhinal cortex (EC). Its alteration compromises communication between the olfactory bulb and the hippocampus, affecting olfactory capacity [8].

The primary cilium is a cellular organelle that senses extracellular signals, which are crucial for the development and function of the nervous system. To avoid abbreviations, we will employ cilia to refer to primary cilia throughout the manuscript. This organelle is a non-motile structure composed of nine pairs of stable microtubules without a central pair, surrounded by a plasma membrane [9]. Cilium is assembled from the mother centriole or basal body, which is constituted by gamma tubulin, pericentrin, and the pericentriolar material (PCM), among other molecules [10,11]. Gamma tubulin functions as the microtubule-organizing center of the cell [12], while pericentrin serves as a multifunctional scaffold for anchoring numerous proteins [10]. Cilia function as a cellular antenna, receiving extracellular signals such as neurotransmitters and neuromodulators through their membrane receptors and transducing these signals intracellularly. Extracellular signaling received by cilia regulates proliferation and disassembly of these structures and mediates differentiation of granule neurons in the cerebellum [13].

Mutations in genes associated with cilia formation and function result in complex neurodevelopmental disorders collectively termed ciliopathies, a group of human inherited genetic disorders (Bardet–Biedl syndrome, Joubert syndrome, etc.) [14]. Ciliopathies are characterized by a diversity of symptoms, such as retinal degeneration, renal disease, and cerebral anomalies, among others, including olfactory impairment (hyposmia) [15]. Previous work has identified olfactory dysfunction by using the University of Pennsylvania Smell Identification Test, detecting a correlation with gray matter volume reduction, as determined by nuclear magnetic resonance imaging [16]. Moreover, olfactory sensory neurons in mouse models of Joubert syndrome ciliopathy have altered responses to odor-evoked electrical potentials and impaired elevation of cytoplasmic Ca^2+^ induced by odors [14]. We have previously reported that human ONPs obtained by exfoliation of the nasal cavity can form cilia in vitro under serum-free conditions. In contrast, ONPs derived from schizophrenia patients display a reduced frequency in cilia formation and a reduction in cilia length [17].

To better understand the mechanisms underlying cilia formation and neuronal differentiation, several studies have explored the effect of specific molecules that modulate these processes. Among them, dibutyryl-cAMP (dbcAMP), a cell-permeable compound, has been shown to promote neuronal differentiation in ONPs [18]. Melatonin (MEL) is a molecule that acts as a neurotrophic factor, modulating proliferation and differentiation in neuronal precursors. These compounds can be valuable tools to stimulate differentiation in ONPs and assess potential defects in patients’ cells under controlled conditions [19]. Given the lack of clear neurological or mechanical causes in these patients, we aimed to investigate whether intrinsic cellular defects might underlie their olfactory dysfunction. In the present study, we have evaluated cilia biogenesis, proliferation, and differentiation abilities in ONPs derived from individuals with hyposmia without neurological deficits or underlying airflow issues (conductive olfactory loss).

## 2. Results

### 2.1. The Combination of Melatonin and Dibutyryl Cyclic AMP Stimulates Maturation and Cilia Biogenesis in Olfactory Neuronal Precursors

**Figure 1:**
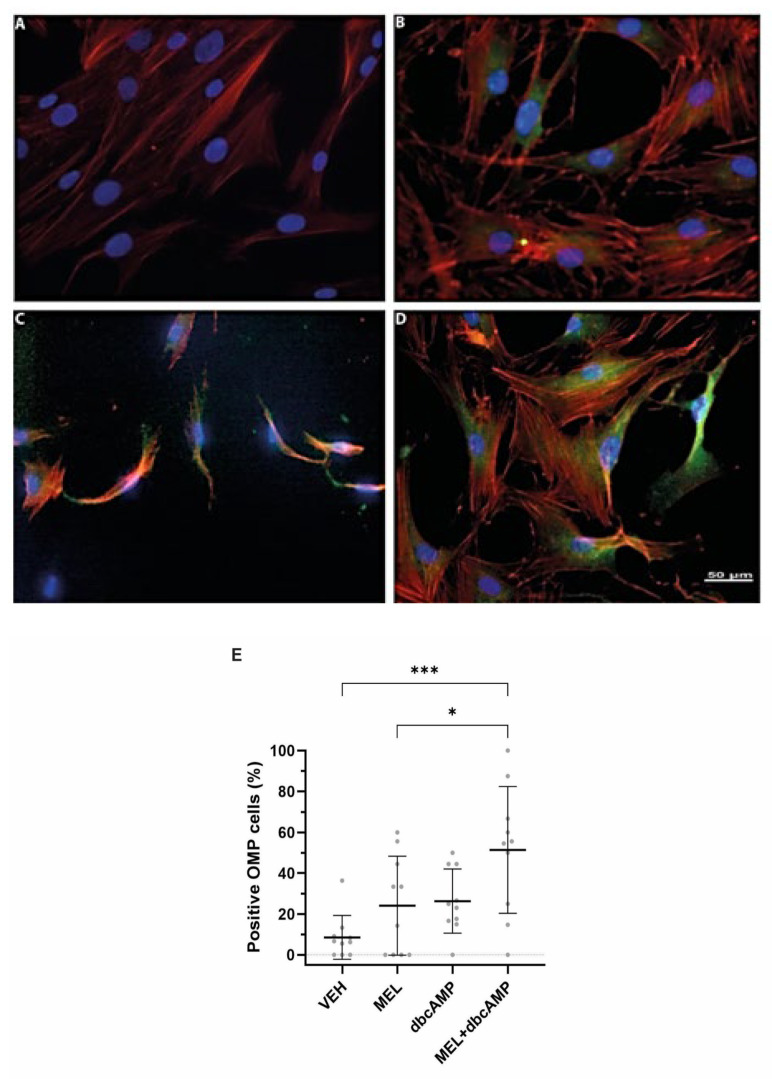
MEL + dbcAMP combination significantly increased the number of OMP-positive cells. Representative images of ONPs treated with (**A**) vehicle (VEH), (**B**) 10 µM melatonin (MEL), (**C**) 2.5 µM dibutyryl cyclic-AMP (dbcAMP), and (**D**) 10 µM melatonin plus 2.5 µM dibutyryl cyclic-AMP (MEL + dbcAMP). ONPs were stained against the anti-OMP marker (green), actin microfilaments were labeled with rhodamine phalloidin (red), and nuclei were visualized with DAPI (blue). (**E**) Scatterplot showing mean ± SD of ONPs positive to OMP (in percentages). Analysis was performed in ten randomly chosen fields. Data analysis was performed by one-way ANOVA corrected for multiple comparisons with Tukey’s test. Statistic differences: * *p* = 0.014 dbcAMP vs. VEH group, and *** *p* = 0.0008, MEL + dbcAMP vs. vehicle.

Many cells assemble primary cilia upon cell cycle exit and enter the G0 phase. In proliferating cells, cilia are a transient organelle assembled in G1 and disassembled prior to mitosis [20]. In this stage of the cell cycle, cells do not proliferate. Instead, they mature and differentiate into a specific cell lineage [21]. ONPs mature to olfactory sensory neurons by expressing key markers such as the olfactory marker protein (OMP). In an earlier study, we demonstrated that 70% of ONPs cultured without serum were in the G0/G1 phase of the cell cycle [17]. Here, we assess neuronal maturation and cilia formation in ONPs upon treatment with (dbcAMP) and MEL. We cultured ONPs from control individuals for 4 days with 10% serum, and at day 5, we added a culture medium without serum and treated them with (a) vehicle (DMEM + ethanol 10 µm, VEH), (b) melatonin 10 µM MEL, (c) dibutyryl cAMP 2.5 µM (dbcAMP), and (d) a combination of MEL 10 µM with dbcAMP 2.5 µM for 120 h (MEL + dbcAMP) [22,23]. In the presence of VEH, a limited number of cells showed immunoreactivity against the OMP marker (Figure 1A). However, an increased number of cells showed OMP staining in response to MEL, dbcAMP, and MEL + dbcAMP treatments (Figure 1B–D). Quantification of the number of OMP-positive cells showed the following (Figure 1E). Cells treated with MEL did not show a significant increase in OMP-positive cells compared to the VEH condition (Figure 1E). Whereas the frequency of OMP-stained cells in the VEH condition represented only 7% of the total cell number, cells incubated with dbcAMP reached 20% and MEL + dbcAMP treatment yielded 50% of OMP-positive cells in the culture (Figure 1E). Notably, we observed a two-fold increase in OMP-positive cells incubated with MEL + dbcAMP when compared with dbcAMP-incubated cells (Figure 1E). These results indicated that the combination of MEL + dbcAMP significantly increased the number of OMP-positive cells, indicating a more mature stage of ONPs.

### 2.2. Cilia Axonemes Are Longer in Olfactory Neuronal Precursors Cultured with Melatonin and Dibutyryl Cyclic AMP

**Figure 2:**
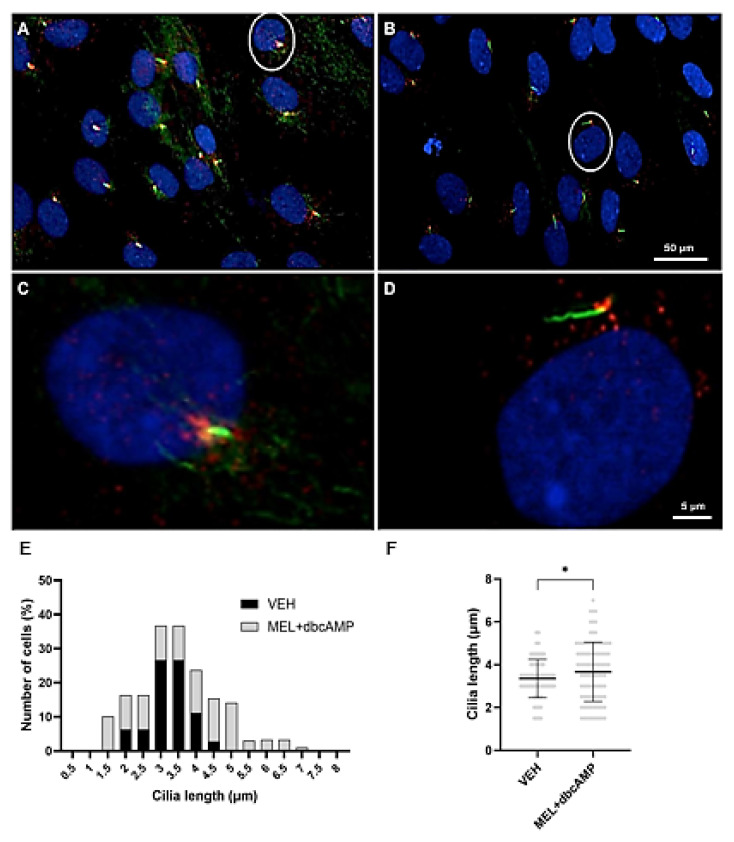
MEL + dbcAMP combination stimulated cilia formation and promoted the assembly of longer axonemes. Confocal fluorescence images of ONPs treated with (**A**) vehicle (VEH) and (**B**) 10 µM melatonin plus 2.5 µM dibutyryl cyclic-AMP (MEL + dbcAMP). ONPs were stained with an anti-acetylated tubulin antibody (green), anti-PCM1 antibody (red), and DAPI (blue) to label nuclei. Higher magnifications of selected ONPs (white circles) from (**C**) VEH and (**D**) MEL + dbcAMP showing primary cilium with axoneme (green) and centrioles (red). (**E**) Histogram showing the frequency distribution of cilia lengths in OPNs incubated with vehicle and MEL+ dbcAMP. Note that the combination triggers cilia formation with cilia lengths ranging from 1.5 to 7 µm. (**F**) Scatterplot showing mean ± SD. Unpaired Student’s *t*-test reveals a significant difference in cilia length between VEH and MEL + dbcAMP treated ONPs * *p* < 0.05.

Previous evidence indicates that the primary cilia protein Arl13b facilitates the maturation of olfactory sensory neurons [24]. Given that we observed that ONPs cultures treated with MEL and dbcAMP increase the frequency of OMP-positive cells in culture, we evaluated cilia biogenesis in ONPs incubated for 120 h with (a) VEH and (b) MEL + dbcAMP (Figure 2A–D). Cilia morphology was determined by measuring the length of the axoneme stained with α-acetylated tubulin, a structural protein of cilia [25]. The more proximal domain of the cilia was identified by PCM-1 staining, a protein primarily localized at the centrosomes, where it helps to maintain cilia structure [26]. Figure 2 shows images of cilia emanating from cultured ONPs from control individuals after VEH (Figure 2A,B) or MEL + dbcAMP treatments, stained with anti-acetylated tubulin and anti-PCM1 antibodies (Figure 2C,D). Our data indicated striking differences in cilia length from ONPs incubated with VEH compared with MEL + dbcAMP (Figure 2B–D). While cilia length in VEH-incubated ONPs was relatively restricted, treatment with MEL + dbcAMP resulted in a broader distribution that included a subpopulation of cells with markedly longer cilia. Remarkably, we detected that 24.8% of ONPs treated with MEL + dbcAMP ONPs developed elongated cilia, in clear contrast with the total absence of these longer cilia with this length in VEH-incubated ONPs (Figure 2B–D). These results indicated that MEL + dbcAMP treatment stimulated cilia dynamics, resulting in the elongation of cilia (Figure 2E,F).

### 2.3. Olfactory Test Results in Control and Hyposmic Subjects

**Figure 3:**
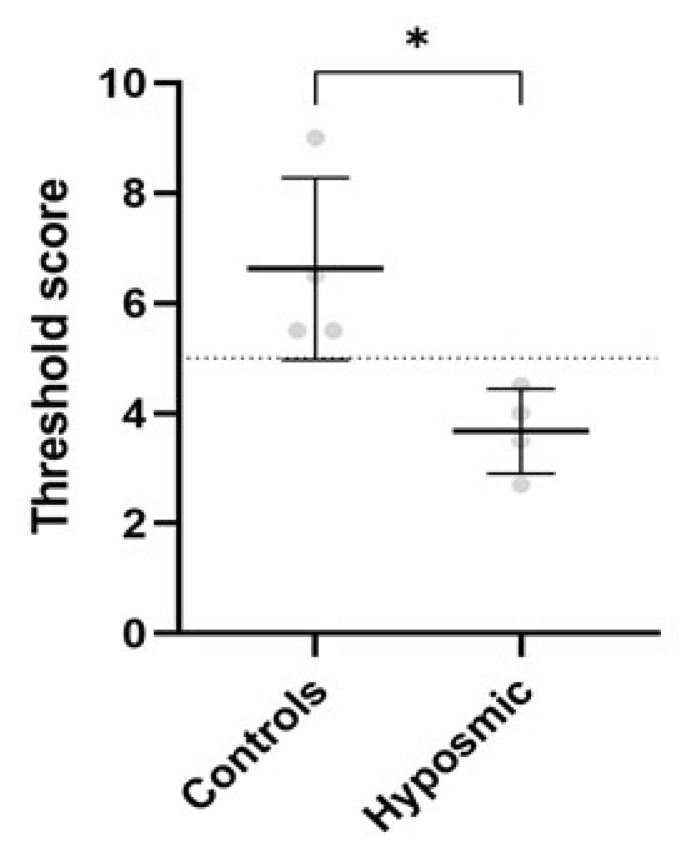
Sniffin’ Sticks threshold test reveals olfactory impairments in hyposmic participants. Scatterplot showing mean ± SD of Sniffin’ Sticks threshold test scores from healthy volunteers (controls; n = 4) and hyposmic individuals (n = 4), n = 8. Unpaired Student’s *t*-test indicates significant differences of * *p* ≤ 0.05 between olfactory performances of control and hyposmic individuals.

Previously, we evaluated the olfactory capacity of both groups using the Sniffin’ Sticks test. The control group (C1–C4) exhibited a mean threshold score olfactory of 6.6 (range: 5.0 to 14.0). In contrast, subjects with olfactory deficits (hyposmia, H1–H4) demonstrated significantly lower values, with a mean of 3.6 (range: 1.0 to 4.9), consistent with hyposmia diagnosis (Figure 3). Statistical analysis using the Mann–Whitney U test confirmed that the difference between groups was significant (*p* < 0.05). Table 1 presents the sociodemographic data and clinical characteristics of all participants, including their olfactory performance scores.

### 2.4. Cilia Are Shorter in Individuals with Hyposmia

**Figure 4:**
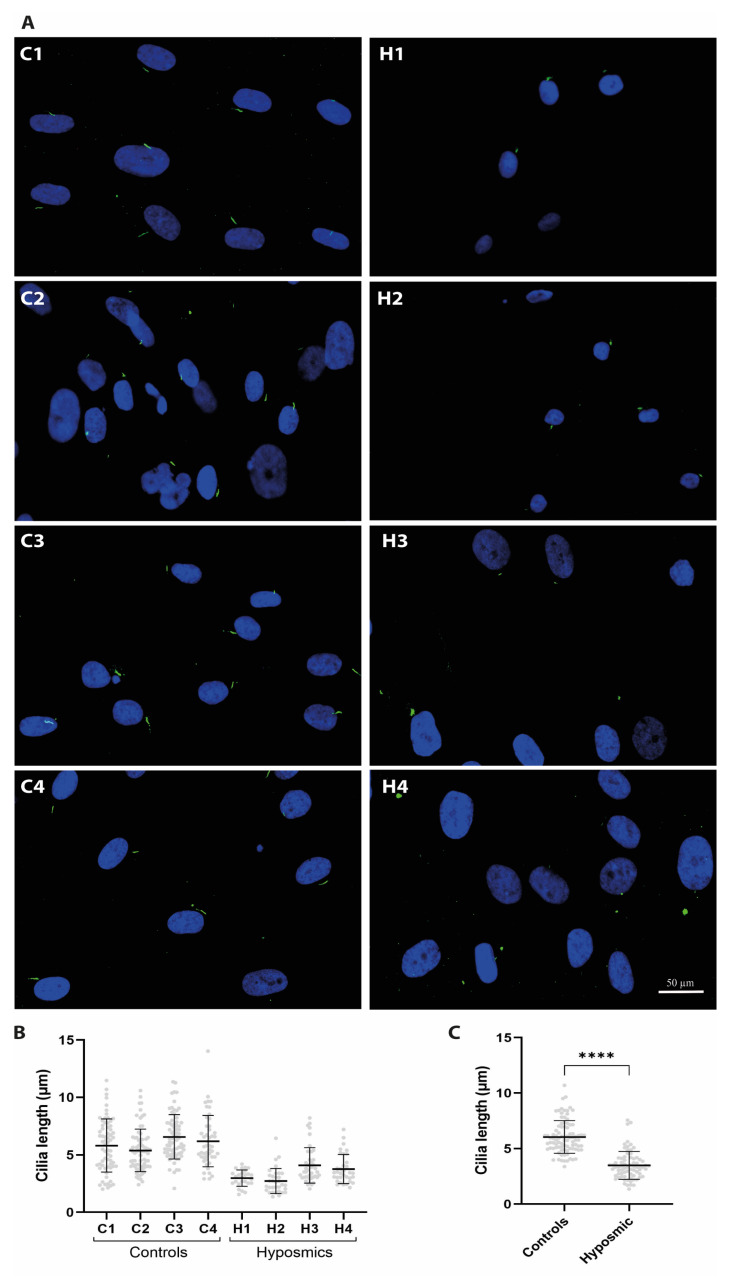
Cilia primary lengths stained with acetylated tubulin in olfactory neuronal precursors ONPs from control and hyposmia subjects. ONPs are treated with 10 µM melatonin (MEL) plus 2.5 µM dibutyryl AMPc (dbcAMP). (**A**) Images are representative of ONPs derived from each participant stained with an acetylated tubulin antibody, followed by a secondary antibody coupled to Alexa 488 (green). Nuclei were stained with DAPI (blue). (**B**) Graph shows the distribution frequency of primary cilia length of ONPs derived from each control (C1–C4) and hyposmia subject (H1–H4), n = 8. (**C**) Cilia length is significantly shorter in hyposmic subjects as compared to controls. Unpaired Student’s *t*-test indicates significant differences, **** *p* ≤ 0.0001.

**Figure 5:**
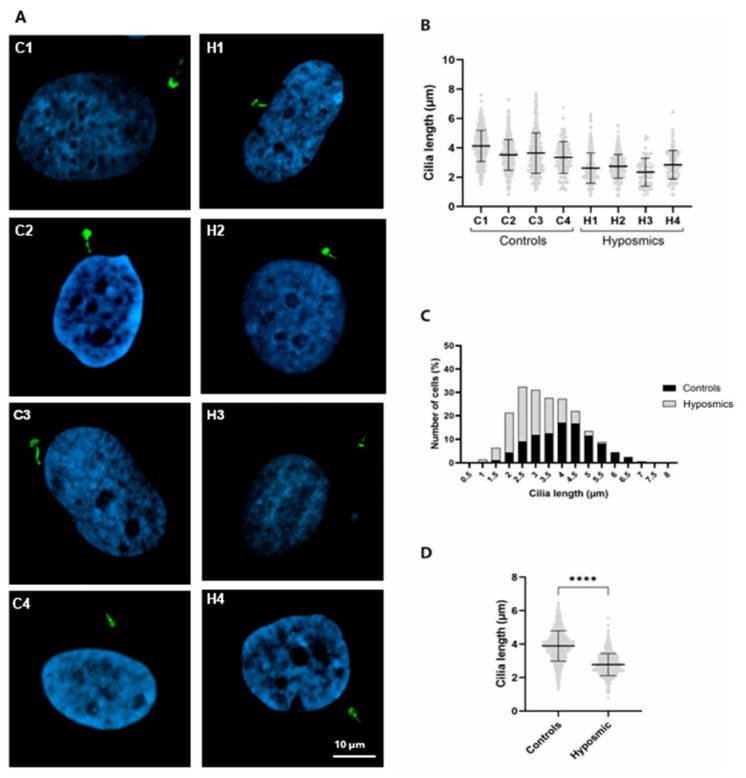
Cilia lengths are shorter in hyposmic subjects. (**A**) Representative confocal images of ONPs from healthy volunteers (C1–C4) and hyposmic individuals (H1–H4). Cilia were stained with an anti-Ar13b and an anti-pericentrin antibody (both in green). Nuclei were stained with DAPI (blue). (**B**) Graphs showing mean ± SD of cilia lengths from all ONPs derived from control (C1–C4) and hyposmic subjects (H1–H4). (**C**) Histogram shows cilia frequency distribution (in percentage) of OPNs from control (C1–C4) and hyposmic (H1–H4) individuals, n = 8. Note the abundance of shorter cilia lengths (from 1.5 to 4.5 µm) in the hyposmic compared to the control group. (**D**) Scatterplot showing mean ± SD of cilia lengths. Unpaired Student’s *t*-test indicates significant differences, **** *p* ≤ 0.0001 between cilia lengths of control and hyposmic individuals.

We obtained ONPs from the olfactory mucosa of young and older healthy volunteers (n = 4, control group) and adults who reported a smell impairment (n = 4, hyposmic group), matched by age and sex, and assessed cilia biogenesis. ONPs from both control and hyposmic individuals were incubated with MEL + dbcAMP and stained with acetylated tubulin-antibody to detect cilia. Images in (Figure 4A) show axoneme staining using α-acetylated tubulin (green) in ONPs from both control and hyposmic groups. Cilia length in controls ONPs ranged from 1.5 to 12 µm, whereas cilia in the hyposmic group ranged from 1 to 7 µm (Figure 4B). We observed longer cilia in ONPs from healthy individuals (C1–C4) compared to subjects with hyposmia (H1–H4) (Figure 4C). Additionally, we stained primary cilia using the ARL13B antibody, which labels the ciliary membrane, to assess their presence and morphology (Figure 5A). Our results reveal striking differences in cilia length between the two groups (Figure 5B). In control individuals, cilia length ranged from 1.5 to 7.0 µm, whereas in participants with hyposmia, the distribution was shifted toward shorter cilia, ranging from 1 to 5.5 µm (Figure 5C). Our data demonstrates that cilia length is significantly altered in subjects with hyposmia, suggesting a potential structural defect in cilia that may contribute to the observed olfactory dysfunction (Figure 5D).

### 2.5. Prtoein Acetylatransferase Activity Hyposmic and Control Participants

Acetylation is required for elongation of cilia [27]; thus, we further investigate possible changes in the acetylation activity of ONPs. We carried out an *N*-acetyl transferase activity assay in ONPs derived from healthy individuals compared to subjects with hyposmia paired by sex (Table 2).

**Figure 6:**
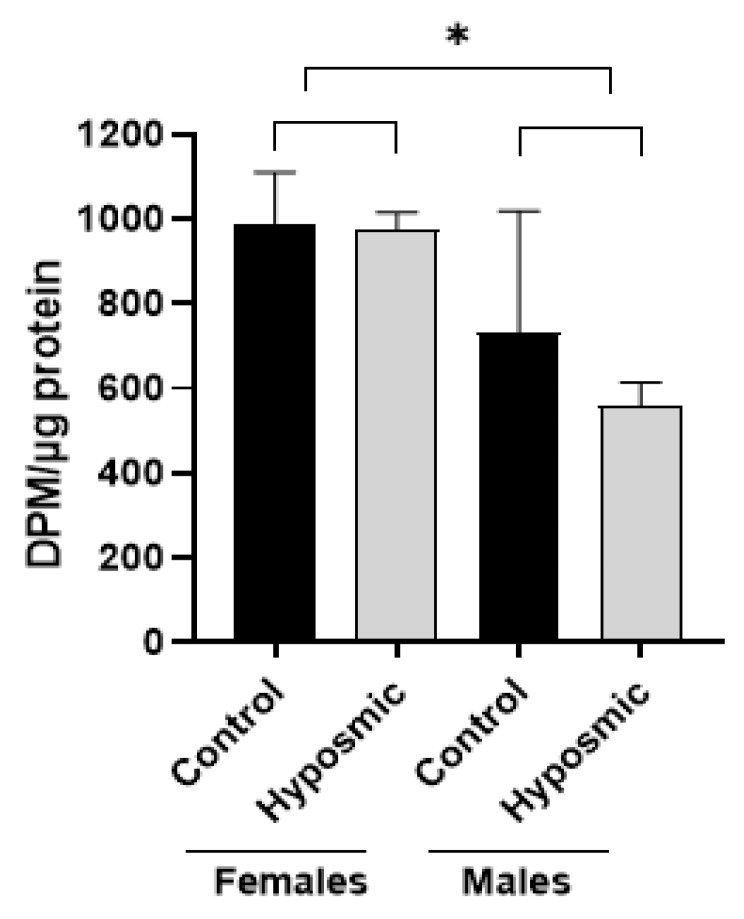
Activity of acetyltransferase is decreased in male participants. * *p* = 0.001.

The two-way analysis of variance (ANOVA) reveals a significant effect of sex (F_(1,12)_ = 17.55, *p* ≤ 0.001), but no differences due to condition or condition × sex interaction. Holm–Sidak post hoc comparisons show that acetylation activity in cells from females was higher than in males in both groups: hyposmia (t = 3.679, *p* ≤ 0.05) and control (t = 2.246, *p* ≤ 0.05). On the other hand, no difference was found between control and hyposmia females (t = 0.0948, *p* = 0.926), even though we detected that ONPs from young males with hyposmia presented lower acetylation activity values (mean= 590.90, DPM/µg, n = 1). However, this difference did not reach statistical significance (t = 1.528, *p* = 0.152). Meanwhile, the two-factor ANOVA revealed that there is a significant difference by sex, F_(1,12)_ = 17.55, *p* = 0.001, and this difference is persistent in both groups, control and hyposmia, and between male and female in both groups: hyposmia (t = 3.679, *p* ≤ 0.05) and control (t = 2.246, *p* ≤ 0.05) (Figure 6).

The graph shows differences between hyposmia and control participants in *N*-acetyl transferase activity of cellular cultures (mean ± standard error of the mean). The two-way ANOVA reveals that there is a significant difference by sex, F_(1,12)_ =17.55, * *p* = 0.001, and this difference is persistent in both groups, control and hyposmia, and between male and female in both groups: hyposmia and control.

### 2.6. Proliferation and Differentiation of Olfactory Neuronal Precursors Are Impaired in Subjects with Hyposmia

**Figure 7:**
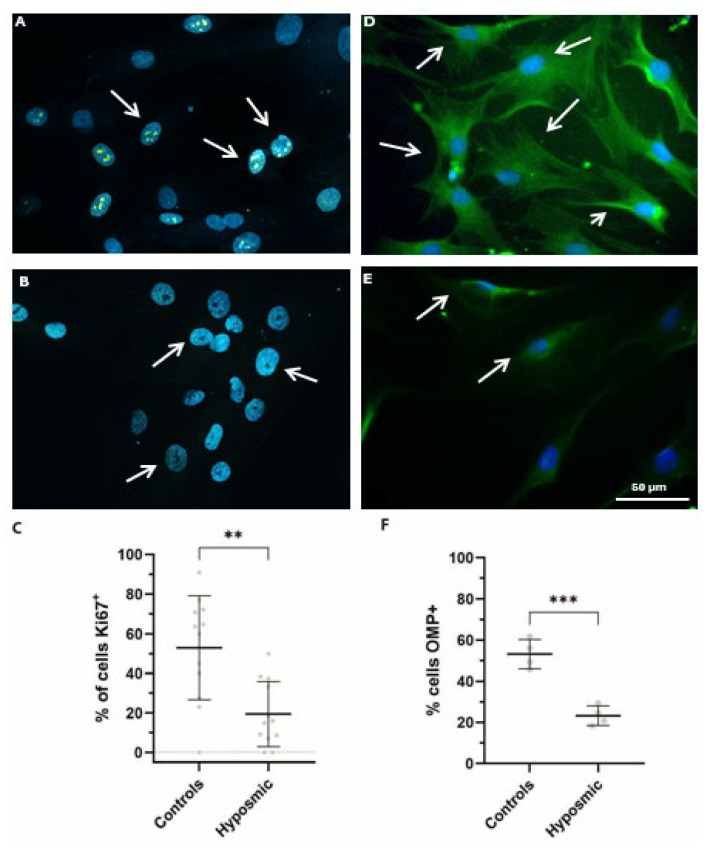
Proliferation and maturation of ONPs are impaired in subjects with hyposmia. Proliferation was assessed using the Ki67 antibody. Representative confocal fluorescent images of ONPs from (**A**) control and (**B**) hyposmic individuals show cells positive for Ki67, indicated by arrows, with nuclei labeled by DAPI (blue). Panel (**A**) displays numerous Ki67-positive cells, whereas panel (**B**) shows only a few. (**C**) Scatterplot shows the mean ± SD (in percentage) of proliferative cells identified by positive Ki67 staining in control and hyposmic subjects. An unpaired Student’s *t*-test indicates a significant difference with ** *p* ≤ 0.01 between Ki67-positive cells in the control group (105 out of 203 cells) and the hyposmic group (33 out of 212 cells). Differentiation was assessed using the OMP marker. Panels display representative images of ONPs from (**D**) control and (**E**) hyposmic subjects stained with anti-OMP antibody (green), indicated by arrows, with nuclei labeled with DAPI (blue). Panel (**D**) shows numerous OMP-positive cells, while panel (**E**) shows only a few. (**F**) Scatterplot shows the mean ± SD of ONPs positive for OMP (in percentages). An unpaired Student’s *t*-test indicates a significant difference of *** *p* ≤ 0.001 between the percentage of OMP-positive cells in the control (52.5 ± 4%) and the hyposmic (26.53 ± 4.8%) groups.

Hyposmia can be triggered by the inability of the neuroepithelial tissue to replace the olfactory neurons from multipotential cells located at the lamina propria [28]. Olfactory tissue regeneration involves both proliferation and differentiation processes [29]. In this study, we assessed the proliferative capacity of ONPs derived from healthy volunteers and individuals with hyposmia. Proliferation was evaluated by immunostaining for Ki67, a nuclear protein widely recognized as a marker of cellular proliferation (Figure 7A–C). Results with Ki67 indicate a notable difference in proliferative activity between groups. In ONPs from healthy volunteers (n = 4), 105 out of 203 cells were positive for Ki67, indicating active proliferation. In contrast, ONPs from participants with hyposmia (n = 4) showed a marked reduction, with only 33 out of 212 cells staining positive for Ki67. We observed statistically significant differences in the proportion of Ki67-positive cells between the control and hyposmic group (*p* < 0.01), suggesting reduced proliferative capacity in ONPs from individuals with hyposmia. (Figure 7C). Additionally, we used an anti-OMP antibody, which targets an abundant cytoplasmic protein in mature sensory neurons (Figure 7D–F). Consistently, when analyzing differentiation rates, we observed 52.5 ± 4.0% OMP-positive cells in ONPs from control individuals (mean ± SD), whereas only 26.5 ± 4.8% were detected in ONPs derived from subjects with hyposmia. The student’s *t*-test yielded a statistically significant *p*-value (*p* = 0.001), indicating a significant difference between the compared groups (Figure 7F). These findings strongly indicate that both proliferation and differentiation processes are impaired in individuals with hyposmia, which could be associated with a diminished ability of ONP to differentiate into mature olfactory neurons.

## 3. Discussion

Cilia dysfunction of olfactory sensory neurons severely impacts smell perception. In addition, chemosensory disturbances like smell and taste disorders are common in the general population, and the incidence of olfactory dysfunction is close to 20%. Importantly, the quality of life of these people is severely reduced, being more prone to suffering a psychiatric disorder [30].

In this pilot study, we demonstrate that a combined treatment of melatonin (MEL) and dibutyryl cyclic AMP (dbcAMP) induces maturation of human-derived cultured ONPs from healthy individuals. The combination of these molecules in cultured ONPs also induces an increase in cilia frequency and cilia length. Our results are in line with previous studies reporting MEL plus dbcAMP having a synergistic effect in enhancing cell differentiation [31]. Indeed, neuronal differentiation of adipose-derived mesenchymal stem cells is increased by using MEL combined with conditioned media from glial cells [31]. On the other hand, the incubation of murine olfactory epithelium cells with dbcAMP and the phorbol ester, 12-*O*-tetradecanoylphorbol-13-acetate, stimulates differentiation into bipolar neurons and promotes the formation of dendritic processes [32]. The mechanism by which melatonin plus dbcAMP promotes cilia formation and controls cilia length is ONPs it is not entirely clear yet, and future studies are necessary to determine the underlying molecular mechanism to these processes. However, cilia formation can be enhanced in human-derived olfactory neuronal precursors when treated with lithium [17]. In addition, lithium treatment induces elongation of primary cilia in the mouse brain and cultured cells, suggesting a potential role in modulating cilia-mediated signaling pathways [33]. In ONPs, lithium, as well as MEL, exerts its effects by inhibiting GSK3-β activity [34], stimulating axonal formation, as reported in hippocampal neurons [35,36]. Moreover, previous findings have demonstrated that cilia formation is regulated by extracellular signals that drive differentiation through transduction pathways involving dbcAMP and G protein-coupled receptors (GPCRs) [32,37]. Our results indicate that cilia frequency increases upon MEL and dbCAMP treatment compared with individual treatments or vehicle conditions, suggesting that the combination of these compounds has an additive effect, leading to a significant increase in cilia formation, which is also associated with elevated expression of the OMP protein, a marker of mature olfactory neurons [18]. All these observations are in line with the scenario of signaling pathways triggered by MEL and involving dbcAMP to enhance cilia formation in cells at G0 stages of the cell cycle. In addition, previous works indicated that MEL promotes proliferation of new neurons in the rat hippocampus through regulation of the cell cycle at phase G2/M [38]. Taken together, these results point towards MEL as a regulator of different processes, differentiation or proliferation, depending upon the stage of the cell cycle. Interestingly, we also observed an increase in cilia length in ONPs following combined treatment with MEL and dbCAMP, suggesting enhanced cilia dynamics. Both compounds are known to promote tubulin polymerization, and elongation of cilia requires the polymerization of soluble tubulin at the axonemal tip. This observation supports the hypothesis that MEL and dbcAMP regulate ciliary dynamics by influencing tubulin polymerization, thereby modulating cilia length [39]. Future studies are warranted to investigate the involvement of tubulin post-translational modifications and intraflagellar transport machinery in cilia length regulation under these treatments.

Moreover, we report that ONPs derived from individuals with hyposmia exhibit less frequent cilia formation, shorter cilia length, and notably a reduced capacity to proliferate and differentiate compared to ONPs obtained from normosmic individuals. These results strongly suggest that there might be an underlying dysfunction in signaling pathways involving MEL and AMPc in the hyposmia clinical context. In this scenario, acetylation of tubulin is a crucial biochemical reaction required for the stability of microtubules that form the axoneme [40]. Therefore, we hypothesized that the reduced cilia formation frequency and length observed in participants with hyposmia could be due to a deficit of acetylation. However, our results showed no significant differences in acetylation between controls and hyposmic participants. Interestingly, we did observe reduced acetylation activity in elder male participants, although conclusions must be taken with caution due to the low sample size of our survey. Additionally, we measured the relative amount of acetylated tubulin, and we found no differences between groups. Abnormalities in the olfactory bulb or injury to any portion of the tract have been described as underlying causes in anosmic or hyposmic patients [41]. Moreover, alterations of proliferation, differentiation, and replacement of olfactory cells have also been related to hyposmia [42]. We speculate that defects in proper cilia formation, together with impaired proliferation and maturation in ONPs, are hindering proper development. Furthermore, the correct replacement of olfactory sensory neurons in the olfactory neuroepithelium ultimately impacts olfactory function. Additionally, we cannot currently rule out that dysfunctional olfactory receptors (ORs) expressed in the olfactory sensory neurons (OSNs) of these patients also contribute to olfaction impairment. Indeed, aberrant expression or dysfunction of OR has been implicated in numerous human diseases, including anosmia [43].

## 4. Materials and Methods

### 4.1. Participants

All participants were examined and diagnosed according to previous guidelines [44]. Subjects with olfactory impairment with no clear etiology were diagnosed as having idiopathic olfactory dysfunction. Participants were adults with the autonomy to sign informed consent, including controls and patients with idiopathic olfactory dysfunction, who were recruited through external consultation to the Instituto Nacional de Psiquiatría Ramón de La Fuente Muñiz (National Institute of Psychiatry Ramón de la Fuente Muñiz), Mexico City. All participants in the study have signed informed consent forms, and the project was approved by the National Institute of Psychiatry Ethics Committee (CEI-010-20170316). Furthermore, this study followed the ethical guidelines set forth by the Declaration of Helsinki to ensure the protection, rights, and well-being of all participants. The participant’s medical history was documented and evaluated, and their olfactory function was assessed using the Sniffin’ Sticks threshold test ODOFIN, Burghart Medical Technology, Wedel, Germany. Four participants diagnosed with idiopathic olfactory dysfunction and four sex- and age-matched control subjects without the disease participated in this pilot study. Sociodemographic data of the participants are shown in Table 1. Clinical histories revealed no medical evidence of neurological illnesses in any of the group under study. At the time of obtaining the samples, participants were receiving different medication regimens as shown in Table 1.

#### Exclusion Criteria

People with chronic rhinosinusitis, allergic rhinitis, post-infectious olfactory dysfunction, nasal surgery, nasal tumors, work exposure to toxic compounds, or those who smoke were excluded from the study. In all cases, individuals without the cognitive capabilities to answer the olfactory threshold questionnaire and participants with topical nasal treatments in the last three months were excluded. 

### 4.2. Olfactory Test

The olfactory capability of the participants was evaluated with a kit of a standard olfactory threshold test containing *n*-butanol (ODOFIN) (Burghart Sniffin’ Sticks Medical Technology, Wedel, Germany). The kit has 16 Sniffin’ Sticks with progressive *n*-butanol dilutions labeled in red (odorized) and 32 Sniffin’ Sticks without odorant labeled in blue and green (non-odorized). The test was applied following the manufacturer’s instructions in a silent and well-ventilated room. A sequence of three Sniffin’ Sticks (2 without and 1 with the odor) was presented to the participant for 3 s each, starting with the highest dilution. The dilution at which the odor was perceived thrice was considered the threshold (critical score 1). The critical score 2 was detected when the participant could no longer perceive the smell of two consecutive lower dilutions. Then, two Sniffin’ Sticks with a higher dilution were presented to the participants, and a critical 3 score was considered when the participants perceived the odor three times. This process was repeated 7 times, and the threshold was the average of the number of dilutions of the Sniffin’ Sticks in which the participant detected and recognized the odor in the last 4 rounds of detection. The scale of the olfactory test range was 0–14. Values are 0–0.9 anosmic, 1–4.9 hyposmia, 5–14 normosmia [45,46].

### 4.3. Obtaining Olfactory Neuronal Precursors and Cell Culture

ONPs were obtained by brushing the nasal cavity with a cytologic brush at the level of the middle turbinate, the medial phase of the lateral wall of the nasal cavity, and the septum, as described in [47]. Cells were mechanically dissociated from the exfoliating brush with a conical device and plated in 24-well cell culture plates. Cells were then cultured in Dulbecco’s Modified Eagle Medium with F-12 (DMEM/F-12; GIBCO, Waltham, MA, USA) supplemented with 10% fetal bovine serum (HI-FBS; GIBCO), 2 mM l-glutamine, 100 µg/mL streptomycin, 100 IU/mL penicillin, Mycozap (Prophylactic 500X, LONZA, Visp, Switzerland), and 5 μg/mL amphotericin. Cells were cultured for 7 days, then detached from the substrate and propagated to passage 3, frozen, and stored in liquid nitrogen, as previously described, until their use [18].

### 4.4. Cilia Formation

ONPs were plated on glass coverslips (~5000 cells/coverslip) in 4-well cell culture plates (Gibco) and cultured for 4 days in DMEM/F-12 supplemented with 10% FBS, glutamine, and antibiotics, as described above. Then, the culture media were changed to DMEM/F-12, with glutamine and antibiotics and without fetal bovine serum. ONPs were treated with either vehicle, 10 µM melatonin (MEL), and 2.5 µM dibutyryl cyclic AMP (dbcAMP), or a combination of 10 µM MEL and 2.5 µM dbcAMP.

Cultures were incubated for 5 days at 37 °C and then fixed with 4% paraformaldehyde in PBS for 15 min, washed three times with PBS, and stored at 4 °C until their processing for immunofluorescence to analyze OMP expression and primary cilium presence. All experiments were performed at passage 5.

### 4.5. Immunofluorescence Staining

Cells fixed as described above were permeabilized with 0.1% Triton X-100 (Santa Cruz, CA, USA) in PBS. After permeabilization, cells were extensively washed and blocked for non-specific binding sites for 1 h with 1% BSA in PBS during 1 h. Then, cultured cells were incubated overnight at 4 °C with the first primary antibodies either for anti-acetylated tubulin (1:200) (Sigma-Aldrich, St. Louis, MO, USA); anti-PCM1 (1:400) (Cell Signaling technology); anti-OMP (1:25) (Santa Cruz, Dallas, TX, USA); anti-Arl13 B (1:1200) (Proteintech, Rosemont, IL, USA); anti-pericentrin (1:5000) (Bethyl, Montgomery, TX, USA); or anti-Ki67 1:100 (Abcam, Cambridge, UK). After washing with PBS, cells were incubated with appropriate secondary antibodies coupled to either Alexa Fluor 532 (4 μg/mL) (Invitrogen, Waltham, MA, USA) Goat anti-rabbit IgG (H+L) Cross-Absorbed, or Alexa 488 or 565 (4 μg/mL) (Invitrogen, Waltham, MA, USA) for 2 h at room temperature. Nuclei were stained with 4′,6-diamino-2-phenylindole (DAPI). Microfilaments were stained with rhodamine–phalloidin coupled to TRITC. Coverslips were mounted in Vectashield (Vector Labs, Burlingame, CA, USA). Cells were observed in an epifluorescence NIKON microscope (Tokyo, Japan), and images were acquired with a Nikon OS-2Mu digital camera or an inverted confocal microscope, LSM900 Carl Zeiss (Oberkochen, Germany).

### 4.6. Determination of Primary Cilia Length and Frequencies for Cell Proliferation and Differentiation

To measure cilia length, the Arl13b or acetylated tubulin was employed to detect the ciliary membrane and the axoneme. Pericentrin labeling was used to detect the base of the cilium. The small GTPase Arl13B is a ciliary protein that regulates the Shh signaling [48], while pericentrin is a scaffold protein that anchors centriolar proteins. This protein forms an integral part of the centrosome and participates in microtubule organization [49]. The length of PC stained with an acetylated tubulin antibody was measured using the NIS-Elements 2.3 software from NIKON (Tokyo, Japan). Images from ONPs stained with antibodies anti-pericentrin, anti-Arl13B and Ki67 were acquired with a confocal microscope model LSM 900 Zeiss that took ten random fields at 63× oil magnification with the tail function, with 6 images around the central image at 7 slices of 4 μm each. The primary cilium was measured with ImageJ Fiji software 1.54 using the Freehand Line tool, marking the base of the cilium to the tip. Furthermore, we also estimated both proliferation and differentiation frequencies in ONPs derived from both groups. Maturation and proliferation frequencies were evaluated with antibodies that recognized protein markers of maturation, olfactory marker protein (OMP), and Ki67 expressed in proliferating cells. The number of cells that showed immunoreactivity for OMP and Ki67 was normalized by the total number of cells/field (considered as 100%) identified with DAPI nuclear staining.

### 4.7. Protein Acetyltransferase Assay

Acetylation levels were assessed by incorporation of [^3^H]Acetyl-Coenzyme A to total proteins. Acetyltransferase reactions were carried out in 20 µL of buffer (Pipes 80 mM pH 6.9, EGTA 1.6 mM, Magnesium sulphate 1 mM, DTT 2 mM), 5 µL GTP 0.4 mM, 20 µg of protein extract in 18 µL of buffer (Pipes 40 mM pH 6.9, EGTA 0.8 mM, magnesium sulphate 0.5 mM, DTT 1 mM), and 10 µL of Acetyl coenzyme-A 20 µM + 0.01 µCi [^3^H]acetyl-CoA (6.8 Ci/mmol). The reaction started with the addition of acetyl coenzyme-A, remained in incubation for 30 min at 22 °C, and was subsequently stopped by spotting 5 µL onto Nylon filters (GE Healthcare, Chicago, IL, USA). Once dried, filters were washed three times in 0.1 M sodium acetate (pH 5.00) and counted in a Beckman Coulter LS 6500 Liquid Scintillation Counter, with Sigma-Fluor (Irving, TX, USA). Universal LSC cocktail (Sigma-Aldrich, St. Louis, MO, USA).

For the protein acetyltransferase assay, the samples were measured in triplicate, and the assay was replicated with two different cell cultures. Two-way analysis of variance was performed with Sigma Stat 3.5 program, setting condition and sex as factors of variation. Pairwise Multiple Comparisons were performed using the Holm–Sidak method, and the significance level *p* ≤ 0.05.

## 5. Conclusions

In this study, we found that neuronal precursors of hyposmia participants have a short primary cilium as well as decreased proliferation and differentiation. Moreover, we found that the combination of MEL plus dbcAMP promotes cilia formation and the enlargement of cilia length in both control and hyposmia participants.

It is essential to highlight that this is the first approach to investigate the correlation between odor perception and cilia morphology and function.

Our results further support the hypothesis that primary cilia defects impair ONP maturation [24,50]. Overall, here we demonstrate that ONPs from subjects with hyposmia exhibit reduced capacities for proliferation and maturation, which can contribute to odor dysfunction since the regeneration of the olfactory neuroepithelium is restricted. Future experiments with larger cohorts of patients are needed to clarify whether the olfactory dysfunction observed in hyposmic patients is due to impaired signaling pathway transduction involving proper cilia formation and function or to defects in ONPs proliferation and differentiation processes.

## Figures and Tables

**Table 1:** Sociodemographic data and clinical characteristics of participants. (control: C1, C2, C3, and C4) and hyposmic individuals (H1, H2, H3, and H4), n = 8. LT: Levothyroxine Smoke; +: positive, -: negative. M: male, F: female. AH: apparently healthy, HD: hypothyroidism disease. The range of the olfactory test scale was 0–14. Range values are: 0–0.9: anosmic, 1–4.9: hyposmia, and 5–14: normosmia.

Participant	Diagnosis	Drug	Diagnosis [Year]	Sample Obtaining	Sex	Age	Olfactory Threshold	Smoke	Concomitant Diseases
1	C1	None	None	2015	F	32	6.5	+	AH
2	C2	None	None	2017	F	37	9.0	-	AH
3	C3	LT	None	2020	M	28	5.5	-	HD
4	C4	None	None	2016	M	73	5.5		AH
n = 4									
1	H1	None	2015	2015	F	32	4.5	-	AH
2	H2	None	2015	2015	F	39	3.5	+	AH
3	H3	None	2015	2015	M	30	4.9	-	AH
4	H4	None	2016	2016	M	88	2.75	+	AH
n = 4									

**Table 2:** Acetylation activity in ONP cultures in control and hyposmic subjects by sex. Female hyposmia (n = 2), male control (n = 2) groups. DPM: Disintegrations per minute. Values are the average of three quantifications from two separate cultures.

Groups	Age	DPM/µg Protein (Mean)
Female control	32	1007.0
		950.6
	37	1142.4
		845.9
Male control	28	1038.8
	73	907.4
		551.0
		430.2
Female hyposmic	28	995.8
		920.2
	39	1011.8
		975.1
Male hyposmic	30	639.3
	88	542.5
		539.7
		512.7

## Data Availability

The datasets generated during and/or analyzed during the current study are available in the [primary cilium database] at [https://psiquiatria-my.sharepoint.com/:f:/g/personal/salarcone_psiquiatria_onmicrosoft_com/Ehuck8KPgnxHt7rhV5rHojsBI-MzdRAHPXmupqT_KORfUQ?e=4tXjwE, accessed on 17 September 2025]. Database of Neuropharmacology Laboratory.

## Abbreviations

The following abbreviations are used in this manuscript:

AD	Alzheimer’s Disease
ANOVA	Two-way analysis of variance
C	Control
DAPI	4′,6-DiAmino-2-PhenylIndole
dbcAMP	dibutyryl cyclic AMP
DMEM	Dulbecco‘s-Modified Eagle Medium
DPM	Disintegrations Per Minute
EC	Entorhinal Cortex
F	Female
GPCRs	G Protein-Coupled Receptors
H	hyposmic
Ki67	Nuclear Protein used as a Marker of Proliferating Cells
M	Male
MEL	Melatonin
OMP	Olfactory Marker Protein ONPs
ONPs	Olfactory Neuron Precursors
OSNs	Olfactory Sensory Neurons
OR	Olfactory Receptor
PCM-1	Pericentriolar Material
PD	Parkinson’s Disease
SD	Standard Deviation
VEH	Vehicle
